# Pushing the limits of immune-related response: a case of “extreme pseudoprogression”

**DOI:** 10.1007/s00262-018-2167-3

**Published:** 2018-05-04

**Authors:** Alvin S. Wong, Yee-Liang Thian, Jeevesh Kapur, Cheng-Nang Leong, Patrick Kee, Chun-Tsu Lee, Martin B. Lee

**Affiliations:** 1 Department of Hematology-Oncology, National University Cancer Institute, National University Health System, NUHS Tower Block level 7, 1E Kent Ridge Road, Singapore, 119228 Singapore; 2 Department of Diagnostic Imaging, National University Health System, Singapore, Singapore; 3 Department of Radiation Oncology, National University Cancer Institute, National University Health System, Singapore, Singapore; 4 Hospice Care Association, Singapore, Singapore; 5 Department of Medicine, National University Health System, Singapore, Singapore

**Keywords:** Immunotherapy, iRECIST, Nivolumab, Pseudoprogression, Renal cell carcinoma

## Abstract

The advent of immune checkpoint targeted immunotherapy has seen a spectrum of immune-related phenomena in both tumor responses and toxicities. We describe a case of pseudoprogression that pushes the limits of immune-related response criteria and challenges the boundaries and definitions set by trial protocols. A middle-aged man with conventional clear cell renal cell carcinoma (RCC) had received multiple prior systemic treatments including vascular endothelial growth factor receptor tyrosine kinase inhibitors, as well as multiple surgeries and radiotherapy treatments. He was eventually started on nivolumab—the anti-programmed death receptor-1 monoclonal antibody approved for the treatment of advanced RCC. Clinical deterioration was observed soon after a 100 mg dose of nivolumab, with onset of acute renal failure and declining performance status. Radiologic progression was documented in multiple sites including worsening tumor infiltration of his residual kidney. The patient was on palliative treatment and visited by the home hospice team in an end-of-life situation. The patient unexpectedly improved and went on to achieve a durable tumor response. The case is illustrative of an extreme manifestation of pseudoprogression, and impels us to probe the assumptions and controversies surrounding this phenomenon.

## Introduction

The advent of immune checkpoint targeted immunotherapy has seen a spectrum of immune-related phenomena related to both tumor response and toxicity. Impressive cases of complete response [1] and abscopal effect [2] in melanoma have been described, and allograft rejection [3] and insulin-dependent diabetes [4] are among the unique adverse events reported. Pseudoprogression [5] is by now a well-known pattern of response and more recently hyperprogression [6] has been described. We report a case of metastatic renal cell carcinoma (RCC) that experienced ‘extreme pseudoprogression’ after a single dose of the anti-programmed death 1 (anti-PD-1) monoclonal antibody nivolumab, with the emergence of a near-death situation soon after treatment. The sequence of events pushes the limits of immune-related response criteria and challenges the boundaries set by clinical trial protocols and the subsequent interpretation of their results.

## Case report

### Pre-immunotherapy history

A 52 year-old Chinese man presented with gross hematuria and had a left nephrectomy done in January 2014. Pathology revealed clear cell RCC of Furhman 2 grading, with invasion of the renal vein and peri-renal fat. There were synchronous solitary right lung and right hilar lymph node metastases. Having declined high-dose interleukin-2, he was started on pazopanib in March 2014, achieving partial response. In January 2015, pazopanib was stopped and surgery was attempted for the oligometastatic disease, but the right hilar node was found to be stuck down intra-operatively. External beam radiotherapy was administered post-operatively in February 2015 to the hilar node (55 Gy in 20 fractions). In May 2015, after 4 months off anti-angiogenic therapy, there was global progression of disease with the right hilar node enlarging and new metastases appearing in multiple sites (lungs, muscle, bones). Sunitinib was started (May to September 2015), and the patient went on to receive further lines of drug treatment with everolimus (October 2015 to February 2016) and axitinib (March to October 2016). He also had palliative surgery to the right radius (curettage and fixation in October 2015) and right proximal femur (curettage and bipolar hemiarthroplasty in November 2015). In November 2015, radiotherapy was also given to the right radius and femur post-operatively, to an enlarging and symptomatic scalp metastasis at the vertex, and to 4 brain metastases by gamma knife technique. Further courses of radiotherapy were given to a large lytic sacral metastasis (February to March 2016), several skin and subcutaneous tumors (May to June 2016), and the left knee (July to August 2016). In addition, subcutaneous denosumab was given as adjunctive treatment for bone metastases from October 2015 to September 2016.

In October 2016, computed tomography (CT) scan showed widespread metastases with interval progression in the skeletal muscles, liver, spleen, right kidney, right adrenal, pancreas, peritoneum, lungs and right hilar nodal mass. Apart from 3 new small cutaneous metastases, the patient did not have symptoms related to any specific organ site. He required the use of a walking aid after his previous hip surgery. Performance status was 2 by Eastern Cooperative Oncology Group score (ECOG). There were multiple enlarging metastases within the single right kidney, but renal function was appropriate for a post-nephrectomy setting. For example, an upper pole lesion now measured 6.5 × 4.3 cm compared to 2.5 × 2.4 cm in the scan 5 months prior. In view of the florid radiologic progression and previous multiple lines of anti-angiogenic treatment, he was offered immunotherapy. Axitinib was stopped and a single dose of nivolumab at 100 mg was given within the same day.

### Post-immunotherapy events

**Fig. 1:**
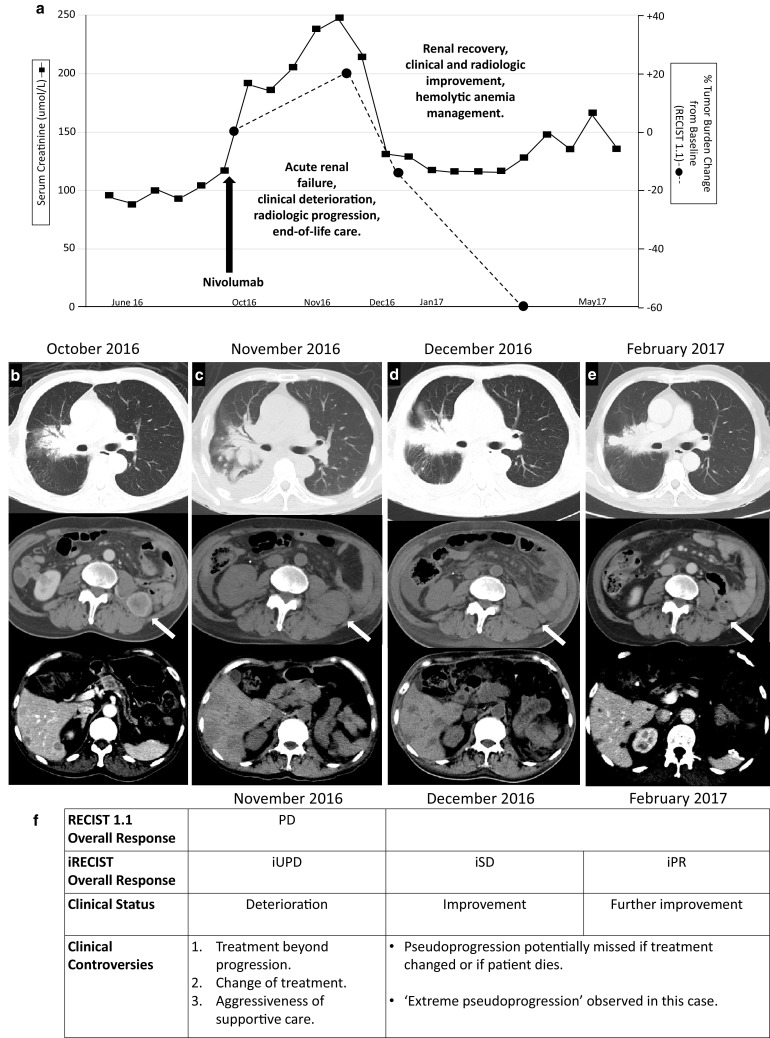
Clinical course. **a** Renal function commensurate with tumor burden. By RECIST 1.1, the percentage change of the sum of target lesions from baseline was + 20, − 13 and − 60%, respectively, in the 3 successive scans taken after nivolumab. Time scale is non-linear. **b** CT scan just before nivolumab. **c** Tumor progression 5 weeks after nivolumab: right hilar mass and lung (top), intramuscular (middle, white arrow), and liver metastases (bottom). **d** Improvement in all sites 11 weeks after nivolumab. **e** Further improvement 20 weeks after nivolumab, with complete resolution of intramuscular metastasis (middle) and bland appearance of residual liver lesions (bottom). Scans in **c** and **d** were without contrast. **f** Tumor assessments by RECIST 1.1 and iRECIST, corresponding clinical status and controversies in clinical decision making or interpretation. *PD* progressive disease, *iUPD* immune unconfirmed progressive disease, *iSD* immune stable disease, *iPR* immune partial response

The patient developed acute renal failure 2 weeks later with oliguria, rising creatinine and hyperkalemia. Serum creatinine rose progressively from a pre-nivolumab level of 117 μmol/L to a high of 247 μmol/L in the following 5 weeks (Fig. 1). Urine sediment was not active (1 white cell and 1 red cell per high power field each, and no casts). Serum phosphate and serum creatine kinase were normal. Other blood indices showed anemia, mild hypercalcemia (2.81 mmol/L), raised alkaline phosphatase (ALP) and lactate dehydrogenase (LDH). Ultrasound (US) of the remaining kidney showed infiltrative renal tumors. There was no hydronephrosis and renal perfusion was good. Except for renal parenchymal tumor infiltration and inflammatory edema, no other plausible causes of acute renal failure were identified. Renal biopsy was not performed as this was a single kidney. There was clinical deterioration with development of dyspnea, back pain, edema and drop in performance status to ECOG 3. At 4 weeks from nivolumab, the subject of hemodialysis was broached but the patient declined. He also expressed his preference not to have aggressive resuscitation or intensive care unit management, and was referred to the home hospice service. Although the recent 3 new skin metastases had resolved, a non-contrasted CT at 5 weeks post-nivolumab documented worsening tumor in multiple sites: skeletal muscles, liver, spleen, pancreas, peritoneal nodules, lungs, right hilar nodal mass and other soft tissue areas. The right kidney was larger, consistent with increasing intrarenal tumor or inflammation. He was at home on expectant management for the next 4 weeks. Several visits were made by the home hospice team. Palliative medications included oral tramadol and gabapentin, and he was prepared for death.

At week 10, the patient unexpectedly walked into the clinic, having felt better a week prior. There was clinical improvement in his general condition and he reported an increase in urine output. Serum creatinine had improved to 131 μmol/L (Fig. 1), ALP was normal, and serum calcium had normalized without any anti-resorptive agent. There was severe anemia (hemoglobin 4.4 g/dL) and the LDH was raised at 1019 units/L (range 250–580). Chest radiography showed improvement in the right hilar and lung shadows. Red cell transfusion was administered. At week 11, non-contrasted CT scan showed improvement in tumor status in most of the involved sites including a decreased size of the right kidney. Blood and bone marrow investigations for the anemia were consistent with immune-mediated hemolysis and oral prednisolone was started at week 13. The patient continued to improve and a contrast CT at week 20 showed dramatic improvement in tumor status. In some sites, including the kidney, essentially complete remission was seen. Serum creatinine returned close to baseline (Fig. 1). Prednisolone was tapered off to complete a 3 month course with hemoglobin stabilizing at 11.3 g/dL. At 6 months post-nivolumab, the patient was doing well without further immunotherapy.

### Renal imaging

**Fig. 2:**
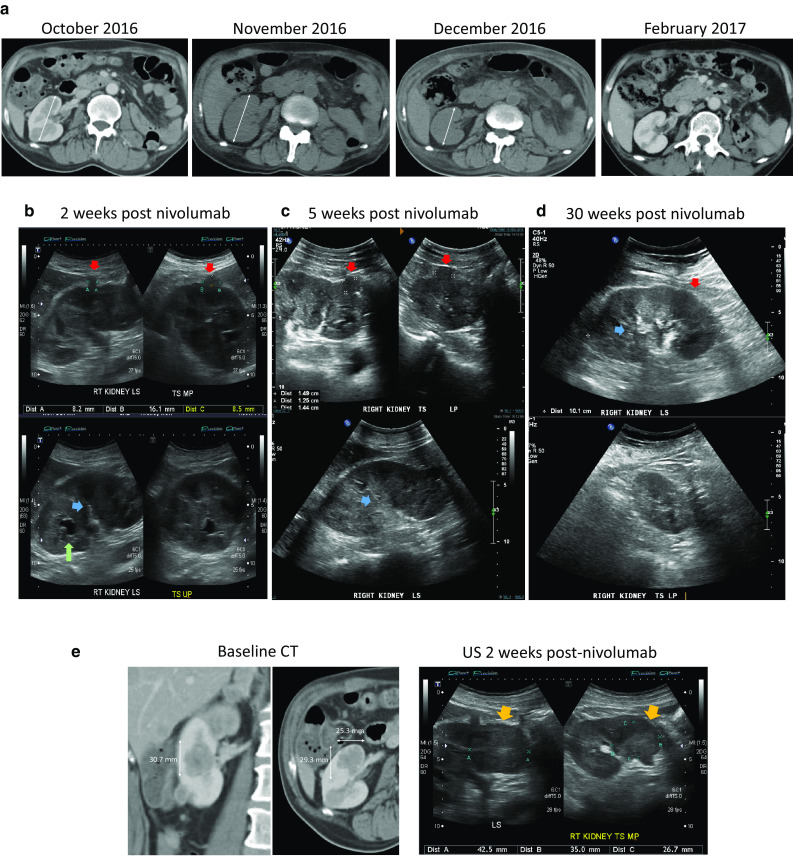
Renal Imaging. **a** Serial CT images with marginal increase in renal size from baseline (October) to 5 weeks after nivolumab (November), marked decrease in renal size at 11 weeks (December) and complete resolution of intrarenal tumors at 20 weeks (February). The changes correspond to the initial deterioration of renal function after nivolumab administration followed by recovery. **b, c** Serial US images during the acute renal failure phase after nivolumab. From week 2 to 5 an enlarging tumor is demonstrated (top, red arrows). There is concomitant increase in cortical swelling with compression and obscuration of the renal medulla and sinus fat (bottom, blue arrows). A renal calyx (bottom, green arrow) seen at week 2 is also subsequently obscured. **d** Corresponding renal US images at week 30, with resolution of renal metastases and cortical swelling, and normal appearance of renal medulla and sinus fat. **e** Increase in a lower pole tumor from baseline CT to the US done at 2 weeks post-nivolumab (yellow arrows)

Serial CT and US images of the right kidney were analyzed (Fig. 2). CT imaging showed marginal increase in kidney size from baseline to the 5 week post-nivolumab scan, and subsequent decrease at the 11 week scan when the renal function had recovered. There was no pre-nivolumab US scan, but the US scans done at 2 and 5 weeks post-nivolumab showed worsening of the renal tumor load (Fig. 2). Onset of diffuse renal cortical swelling was also noted in the US at 5 weeks post-nivolumab, as demonstrated by the progressive compression and obscuration of renal medulla and sinus fat. The US changes are commensurate with the progressive worsening of renal function at these time points. A lower pole metastasis shown in the US at 2 weeks post-nivolumab was significantly larger than the corresponding lesion on the baseline contrast CT, despite the differences in imaging modality. A contrast CT at 4 months as well as an US at 6 months post-nivolumab showed decreased renal size and near complete resolution of the renal metastases.

## Discussion

Pseudoprogression is a known phenomenon of immune checkpoint inhibitor therapy, and has been variably defined as a response after an initial increase of tumor burden, a reduction in tumor burden during or after the appearance of new lesions, or an increase in tumor burden not confirmed as progressive disease at the next imaging assessment [7]. Conceptually, pseudoprogression refers to an initial progressive disease by conventional Response Evaluation Criteria in Solid Tumors (RECIST) criteria, but with subsequent improvement that may be a durable response [7–9]. The phenomenon could be explained by an initial influx of immune or inflammatory cells with or without edema causing enlargement of tumor, or by continued tumor growth preceding a delayed effect of the immune system [10].

This case demonstrated initial increase in tumor burden with associated clinical instability, critical organ insufficiency and expected death. At 5 weeks, the first response assessment by RECIST would have been ‘progressive disease’ (Fig. 1), although the introduction of immune-RECIST (iRECIST) criteria [8] would have qualified for ‘immune unconfirmed progressive disease’ (iUPD), with the subsequent two scans improving to ‘immune stable disease’ (iSD) and ‘immune partial response’ (iPR), respectively. Given the initial clinical deterioration, standard clinical practice would have called for a decision not to treat beyond progression, to abandon study treatment if the patient was on trial protocol, and to change treatment if the patient was fit enough for more treatment. An additional controversy would have been the dubious recommendation for intensive supportive care such as hemodialysis or other life-sustaining measures. With the hindsight that this patient’s condition improved after the initial crisis without intervention, one could say that in another case pseudoprogression would be potentially missed if a new line of drug treatment was instituted, or if the patient died from an organ failure that could have been supported with aggressive means.

Accelerated tumor growth rate following the cessation of anti-angiogenic therapy has been observed in both animal models [11] and human cases of metastatic cancer [12]. This could well account for the initial rapid progression of disease we saw in this case. However, the definition of pseudoprogression is a clinical one in which initial progression may be due to either tumor growth or immune infiltrate, with both possible mechanisms supported by biopsy evidence [10]. If anything, the cessation of axitinib just prior to the point of starting nivolumab could have been a trigger to the dramatic sequence of events, which are consistent with and strikingly illustrate the clinical phenomenon of pseudoprogression.

The development of immune-RECIST criteria attempts to allow for the phenomenon of pseudoprogression [5, 7, 8], while the decision to continue the same treatment beyond RECIST defined progression is dependent on a multiplicity of factors processed via an individualized approach [13]. For example, in various Checkmate trials, the criteria to treat beyond progression was tolerability and apparent clinical benefit, assessed at the discretion of the investigator [14, 15]. The presumption is that only those with favorable clinical characteristics at progression would merit continuing the same treatment instead of changing to another. Clinical decompensation ordinarily leads to treatment decisions that would obviate the possibility of subsequently observing the evolution of pseudoprogression. This unique case demonstrates that clinical deterioration is not an exclusion to the phenomenon, and raises several provocative questions. Should patients be treated beyond progression only if there was clinical stability? Should patients who progress initially and become unstable be aggressively supported in the hope that there would be a subsequent improvement? Are there clinical indicators or biomarkers that can guide decision making in this situation [7]? Are the low reported pseudoprogression rates [5] in the literature an underestimation of this phenomenon?

We propose that ‘*extreme* pseudoprogression’ be a term that describes the scenario where pseudoprogression is accompanied by initial clinical deterioration or instability, even to a life-threatening degree, with subsequent clinical improvement and tumor response. It would be interesting to see if cases with ‘extreme pseudoprogression’ also have prolonged survival durations as do pseudoprogression cases in general [5, 10].

We continue to be bemused by the spectrum of immune-related phenomena seen in clinical practice. This single case of ‘extreme pseudoprogression’ provokes us to re-examine our approach to immune-related response and clinical trial interpretation.

## Abbreviations

ALP: Alkaline phosphatase
CT: Computed tomography
ECOG: Eastern Cooperative Oncology Group
iPR: Immune partial response
iRECIST: Immune-RECIST
iSD: Immune stable disease
iUPD: Immune unconfirmed progressive disease
RCC: Renal cell carcinoma
US: Ultrasound
